# Transgenic Rabbits That Overexpress the Neonatal Fc Receptor (FcRn) Generate Higher Quantities and Improved Qualities of Anti-Thymocyte Globulin (ATG)

**DOI:** 10.1371/journal.pone.0076839

**Published:** 2013-10-23

**Authors:** Mária Baranyi, Judit Cervenak, Balázs Bender, Imre Kacskovics

**Affiliations:** ImmunoGenes Kft, Budakeszi, Hungary; University Paris Sud, France

## Abstract

Immune suppression with rabbit anti-thymocyte globulin (rATG) is a well-established therapeutic concept for preventing host rejection of transplanted organs and graft versus host disease. Increasing the efficiency of rATG production by reducing the number of animals would be highly beneficial to lower cost and to improve quality standards. We have developed transgenic (Tg) mice and rabbits that overexpress the neonatal Fc receptor (FcRn) and have shown an augmented humoral immune response in these animals. To test whether our FcRn Tg rabbits produced rATG more efficiently, we immunized them and their New Zealand White controls with live Jurkat cells. By day 21 after immunization, Tg animals produced significantly, 1.5 times higher amount of total IgG compared to their wt littermates. Also, the binding efficiency of Tg sera to Jurkat cells and their complement-mediated cytotoxicity was significantly higher. The purified Tg IgG preparation contained 2.6 the amount of Jurkat specific IgG as the wt preparation analyzed by complement-mediated lysis, suggesting greater antigen-specific B cell activation in the Tg rabbits. To test this hypothesis, immunization with ovalbumin and human α_1_-antitrypsin was performed, resulting in significantly greater numbers of antigen-specific B-cells in the FcRn Tg rabbits as compared with wt controls. The shift towards significantly larger populations of antigen-specific B cells relative to the non-specific B cell pool is further corroborated by our previous findings in FcRn Tg mice. Consequently, our FcRn Tg rabbits have the potential to offer substantial qualitative and quantitative improvements for the production of rATG and other polyclonal or monoclonal antibodies.

## Introduction

Polyclonal ATG - the purified IgG fraction of serum from rabbits, horses, or less commonly goats immunized with human thymocytes or the Jurkat T-lymphoblastic cell line - contains antibodies with a wide range of specificities against antigens expressed on various normal and malignant hematopoietic cells including T-, B-, NK, and dendritic cells [1]. Due to its immunosuppressive potency, ATGs are extensively used in clinical applications, mainly in the field of human transplantation. These applications include therapy of aplastic anemia [2], conditioning of recipients of bone marrow transplantation [3], [4], treatment of graft-versus-host disease after bone marrow transplantation [5], [6], and prevention and treatment of acute rejection of organ allografts, including steroid resistant rejection [7]. There has been growing evidence that ATGs have potent cytotoxic effects particularly on lymphatic and to a lesser extent on myeloid malignancies [8]. The mechanisms involved in the cytotoxic effects include complement-dependent cytolysis, cell-mediated antibody-dependent cytotoxicity, opsonisation and subsequent phagocytosis by macrophages, activation-induced cell death as well as apoptosis [9]–[12]. There is also evidence that ATG-mediated immunosuppression is delivered in part via immunologically specific actions involving the generation of regulatory T cells [13], [14].

Currently, there are three commercially available preparations of ATG. ATG-Fresenius® (Fresenius-Biotech GmBH) is produced by immunization of New Zealand White (NZW) rabbits with the Jurkat human T-lymphoblastic cell line. Thymoglobulin® (Genzyme) is produced by immunizing NZW rabbits with human thymocytes while ATGAM® (Pharmacia Upjohn) is produced by immunizing horses with human thymocytes. Due to differences in manufacturing procedures, the different types of ATGs contain variable specificities and amounts of antibodies explaining the wide variability in doses used in the clinical setting. For Thymoglobulin, a total recommended dose is 4.5 to 8 mg/kg body weight for matched unrelated donor allogeneic hematopoetic stem cell transplantation; for ATG-Fresenius the recommended dose for the same indication is about ten times as high [15]. As demand for ATG has grown, producers have expanded their facilities to house hundreds of thousands of rabbits in costly high-standard facilities under specified pathogen-free (SPF) conditions. In addition, commercial manufacturers are trying to solve the challenge of producing sufficient amounts of ATG by vaccination of larger animals such as horses where SPF environments cannot be established and thus the percentage in antigen specific antibodies is relatively low (due to high baseline IgG levels).

Increasing the efficiency of rabbit (r)ATG production and reducing the number of animals would be highly beneficial to maintain consistent quality standards. It would also lower costs and reduce the ethical concerns related to keeping large animal colonies for rATG production.

The use of genetically modified animals is widely accepted for the production of therapeutic monoclonal antibodies and remains under investigation for the production of polyclonal antibodies [16]. We were interested to study the benefits for rATG production by using our transgenic (Tg) rabbits that overexpress the rabbit neonatal Fc receptor (FcRn) and that show a much augmented humoral immune response [17]. In this study, we immunized the rabbit FcRn Tg rabbits and their wild type (wt) littermates with human lymphoblast T-cell line cells (Jurkat cells) and analyzed their immune sera with binding and complement-dependent cytotoxic assays to compare their ATG production efficiency.

Our data indicate that the sera of the Tg rabbits have greater binding and cytotoxic activity and that their purified IgGs contain proportionally higher amount of antigen-specific IgG. The higher specific activity of IgG harvested from FcRn overexpressing animals is highly valuable as lower doses of purified IgG are needed for therapy. In addition, they are harvestable from fewer animals and therefore reduce consistency problems and exposure to non-antigen specific rabbit IgG.

Our previous studies with Tg mice that overexpress FcRn indicated that these animals produce greater number of antigen specific B cells as compared to their wt controls. Thus, we decided to analyze the number of antigen-specific B-cells of these FcRn Tg rabbits by immunizing them with soluble proteins and analyzing the number of antigen-specific B-cells with ELISPOT assay. The results of these studies confirmed that FcRn overexpression in these Tg rabbits enhances the expansion of antigen-specific B cells and plasma cells, similarly to what we observed in the immunized bFcRn Tg mice.

This study shows that Tg rabbits that overexpress FcRn produce 1.5 times as much total IgG after immunization with Jurkat cells as their wild type controls. The proportion of rATG in the Jurkat-induced antibody pool is 2.6 times than that of the control rabbits. These findings offer major improvements for production of rATG in animals.

## Materials and Methods

### 1. Animals, housing and ethics statement

Tg rabbits that have enhanced FcRn activity because they carry and express one extra copy of the rabbit FcRn α-chain encoding gene (rabbit FCGRT) in addition to the endogenous rabbit FCGRT gene on New Zealand White rabbit genetic background are coded as NZW Tg1 rabbit FCGRT (78) wherein 78 refers to the founder line [17]. These Tg rabbits and their wild type (wt) littermates were housed in specified pathogen free facility.

The treatments of rabbits in this study were carried out in strict accordance with the recommendations in the Guide of the Institutional Animal Care and Ethics Committee at ImmunoGenes Kft that was approved with permissions 22.1/601/000/2009 and XIV-I-001/2086-4/2012 issued by the Food Chain Safety and Animal Health Directorate of the Government Office of Pest County, Hungary.

### 2. Immunizations

#### Preparation of anti-thymocyte serum

Jurkat E6.1 T cells obtained from the European Collection of Cell Cultures (Salisbury, UK) were used as antigen. Before immunization, Jurkat cells were harvested, washed twice with phosphate buffered saline (Sigma-Aldrich, Budapest), and used as a suspension at a concentration of 1×10^8^ cells per ml. Ten Tg and 10 littermate wt rabbits received two successive intravenous injections 14 days apart of 5×10^8^ living Jurkat T cells, as was suggested in earlier reports, indicating that longer courses of injection usually yields less effective antisera [18], [19]. Blood samples were collected before the first immunization and on days 7, 14 and 21 and Jurkat-specific antibodies were analyzed by binding studies using flow cytometry based on previous report [20]. Sera were prepared by centrifugation, heated to 56°C for 30 minutes, and stored at −20°C. Normal rabbit serum as complement source for cytotoxicity assays was obtained from non-immunized NZW rabbits and processed in an identical manner but without heating.

#### Immunization with soluble antigens

Ovalbumin (OVA) immunization: 7 Tg and 7 wt rabbits were immunized subcutaneously with 300 µg OVA (Sigma-Aldrich, Budapest) in complete Freund's Adjuvant (CFA) and challenged 21 days later with 150 µg OVA in incomplete Freund's Adjuvant (IFA). Human α_1_-antitrypsin (hA1AT) immunization: 10 Tg and 4 wt rabbits were immunized subcutaneously with 300 µg hA1AT (Sigma-Aldrich, Budapest) in CFA and challenged 21, 42 and 63 days later with 150 µg antigen in IFA.

### 3. Protein G-agarose chromatography for IgG purification

Serum samples of Tg and wt rabbits were pooled, diluted with binding buffer (0.2 M Na-phosphate buffer, pH 6.0) and bound to Protein G agarose beads (Pierce Protein G Plus Agarose, Thermo Scientific) by incubation for 30 minutes at room temperature with slow agitation. IgG was eluted using 0.1 M Na-citrate buffer (pH 2.9), dialyzed against PBS, aliquoted and stored at −20°C until use.

### 4. Flow cytometry

Flow cytometry measurements were carried out using fluorescence-activated cell sorting (FACS) on a FACSCalibur cytometer (BD Biosciences, San Jose, CA, USA). Results were analyzed using FCS Express Version 3 software (De Novo Software, Los Angeles, CA, USA).

#### Binding assay

Dilutions of the individual sera and Protein G purified pooled IgG from the Jurkat-immunized Tg and wt rabbits were prepared in FACS buffer (phosphate-buffered saline complemented with 1% fetal bovine serum and 0.04% azide). 5×10^5^ Jurkat cells per sample were washed with FACS buffer, then 100 µl diluted immune serum was added and incubated on ice for 30 minutes. After washing with FACS buffer, cell bound antibodies were detected by incubation with 30 µl of 1000-times diluted DyLight™ 649-conjugated AffiniPure goat anti-rabbit IgG (Jackson ImmunoResearch Laboratories, Inc., West Grove, PA, USA) for 30 minutes on ice. Results were visualized by plotting the mean fluorescence intensities (MFI), which were measured at the same instrumental setting for all samples. Serial dilutions of the purified IgG samples were applied and antibody cell binding was determined by GraphPad Prism version 5 for Windows (GraphPad Software, La Jolla California USA) using nonlinear regression - one site binding (hyperbola) algorithm.

#### Cytotoxicity assay

Complement-mediated cytotoxicity was performed as follows: 1×10^6^ Jurkat cells per sample were washed with FACS buffer and incubated for 30 minutes at 37°C with 100 µl of diluted heat inactivated individual immune sera or total IgG purified from pooled Tg and wt immune sera, and 10 µl normal rabbit serum as complement source. Cytotoxicity was determined by propidium iodide (PI) incorporation. The number of lysed cells from the cytotoxicity of the immune serum samples were determined by comparison with the negative control samples (with added complement source without immune serum). ATG-Fresenius S was used as reference and all samples were measured at the same instrumental setting. Serial dilutions of purified IgG samples were applied and complement mediated cytotoxicity was determined by GraphPad Prism version 5 for Windows (GraphPad Software, La Jolla California USA) using nonlinear regression - one site binding (hyperbola) algorithm.

#### Analysis of spleen cell populations

Single-cell suspensions from OVA and hA1AT immunized rabbit spleens were isolated and incubated with FITC-coupled mouse anti-rabbit T lymphocyte (AbD Serotec) and RPE-coupled goat anti-rabbit IgM (Southern Biotech) antibodies using standard protocol. Isotype controls were obtained from BD Pharmingen. Proportion of granulocytes was estimated based on forward scatter/side scatter dotplots.

### 5. ELISA measurements of antigen specific and total immunoglobulin levels

High binding ELISA plates (Corning Inc., NY, USA) were coated with 5 µg/ml OVA, 5 µg/ml hA1AT or 1 µg/ml unlabeled goat anti-rabbit IgG (H+L) (Southern Biotechnology Associates Inc., Birmingham, AL, USA) in 0.1 M sodium carbonate-bicarbonate buffer (pH 9.6) for 2 hours at room temperature (RT) or overnight a 4°C, washed with 0.1 M phosphate-buffered saline (PBS, pH 7.2) containing 0.05% Tween-20 (PBS-T) and blocked with PBS containing 1% BSA for 1 hour at RT. Diluted serum samples were added to the wells (in independently diluted triplicates) and incubated for 1 hour at RT. In case of total IgG measurement each plate included standard controls of serially diluted, purified rabbit IgG (in duplicates). After washing, bound serum antibodies were detected by horseradish peroxidase labeled goat anti-rabbit IgG (Southern Biotechnology Associates Inc., Birmingham, AL, USA) using tetramethyl-benzidine (TMB, Sigma-Aldrich Co., St. Louis, MO, USA) as chromogen. Blank corrected optical density at 450 nm was measured with a Multiscan ELISA Plate Reader (Thermo Electron Corporation, USA) and interpreted using ScanIt Software 2.5.1 Research Edition for Multiscan FC (Thermo Fisher Scientific, Vantaa, Finland). For data analysis, GraphPad Prism Version 5 for Windows software (GraphPad Software, La Jolla, CA, USA) was used. Serum IgG concentrations were interpolated from the linear portion of the standard curve, based on the blank-corrected absorbance values using the one site binding hyperbola function of non-linear regression curve fit. IgG titers are given as half-maximal values (inflexion point titer, indicated as “titer”) or as dilutions at an optical density of 0.05 (endpoint titer).

### 6. ELISPOT assays

MultiScreen HTS plates (Millipore, Bedford, MA) were coated with 100 µg/ml OVA, or hA1AT, respectively in PBS, at room temperature for 3 h. The plates were then washed with PBS and blocked with RPMI 1640 medium containing 5% FCS and mercaptoethanol (50 mM) for 30 min at room temperature. Serial dilutions (starting at 5×10^5^ cells/well) of spleen lymphocytes harvested on day 26 of OVA and day 70 of hA1AT immunizations were added to the wells. The plates were incubated at 37°C with 5% CO_2_ overnight and washed with PBS-T; HRP-conjugated goat anti-rabbit IgM and IgG (1∶4000-fold dilution; Southern Biotechnology Associates) was then added to each well. After 1 h incubation at room temperature, the plates were washed with PBS-T. The plates were then incubated in the presence of a chromogen, 3-amino-9-ethylcarbazole (Sigma-Aldrich), and H_2_O_2_ as substrate at room temperature, and the reaction was terminated by a water wash. The spots were counted in the ImmunoScan ELISPOT reader (Cellular Technology) and evaluated by ImmunoSpot software version 3.2 (Cellular Technology).

### 7. Statistical analysis

Statistical differences were calculated by pair-wise comparisons of relevant groups using permutation tests. Briefly, values from the groups to be compared were randomly reassigned to two groups and the difference between the group means was determined. Distribution of 5000 randomizations was drawn and the two-tailed *P*-value corresponding to the real sample assignments was calculated. The arithmetic mean of 50 such *P*-values was accepted as the probability of α-error. Values of *P*<0.05 were considered significant and were indicated as follows: *, *P*<0.05; **, *P*<0.01; ***, *P*<0.001.

## Results

### 1. FcRn Tg rabbits produce higher level of total IgG after live Jurkat cell immunization

**Figure 1:**
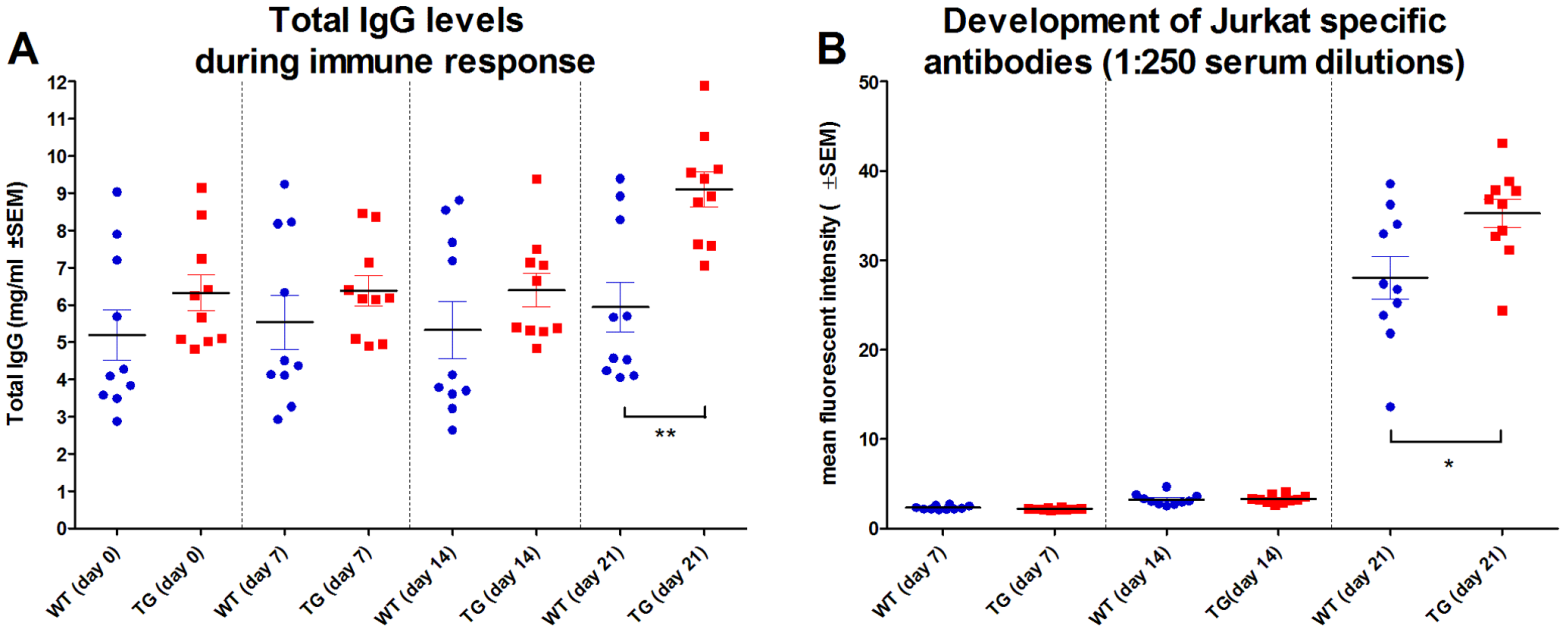
Kinetics of Jurkat specific immune response in Tg and wt rabbits. **A.** Total IgG concentrations of serum samples collected from rabbit FcRn transgenic (Tg) and wild type (wt) rabbits immunized with Jurkat T cells (determined by ELISA assay). **B.** Development of immune response to Jurkat T cell surface antigens during immunization (determined by flow cytometry binding assay of 1∶250-fold diluted individual rabbit serum samples). Each dot represents one animal as an average of three measurements; (* *P*<0.05; ** *P*<0.01).

Antisera were raised by immunizing Tg rabbits and their wild type siblings with two successive intravenous injections 14 days apart of a single cell suspension of 5×10^8^ living Jurkat cells and blood samples were collected before the first immunization and on days 7, 14 and 21. Total IgG contents of individual rabbit immune sera were measured from all collected blood samples. During the first 14 days none of the rabbits showed significant increase in total IgG content compared to day 0 (wt = 5.20±2.14 mg/ml, Tg = 6.32±1.51 mg/ml). As observed in our previous experiments, Tg rabbits had slightly, though not statistically significantly, higher total IgG levels even before immunization. By day 21, Tg animals had significantly, 1.5 times higher (*P* = 0.0016) total IgG as compared to their wt littermates (9.10±1.46 mg/ml and 5.95±2.11 mg/ml, respectively) (**Figure 1A**).

### 2. FcRn Tg rabbits produce higher amount of Jurkat cell specific antibodies

Antibody levels of collected immune sera raised against Jurkat cell surface antigens were measured by flow cytometry using individual Tg and wt samples, initially at 1∶250-fold dilution. Prior to day 14 no significant antibody production could be detected. By day 21 a strong immune response was measured in both groups, with those of the Tg rabbits being significantly higher in Jurkat specific IgG titers (*P* = 0.0216) compared to the wt animals (**Figure 1B**).

**Figure 2:**
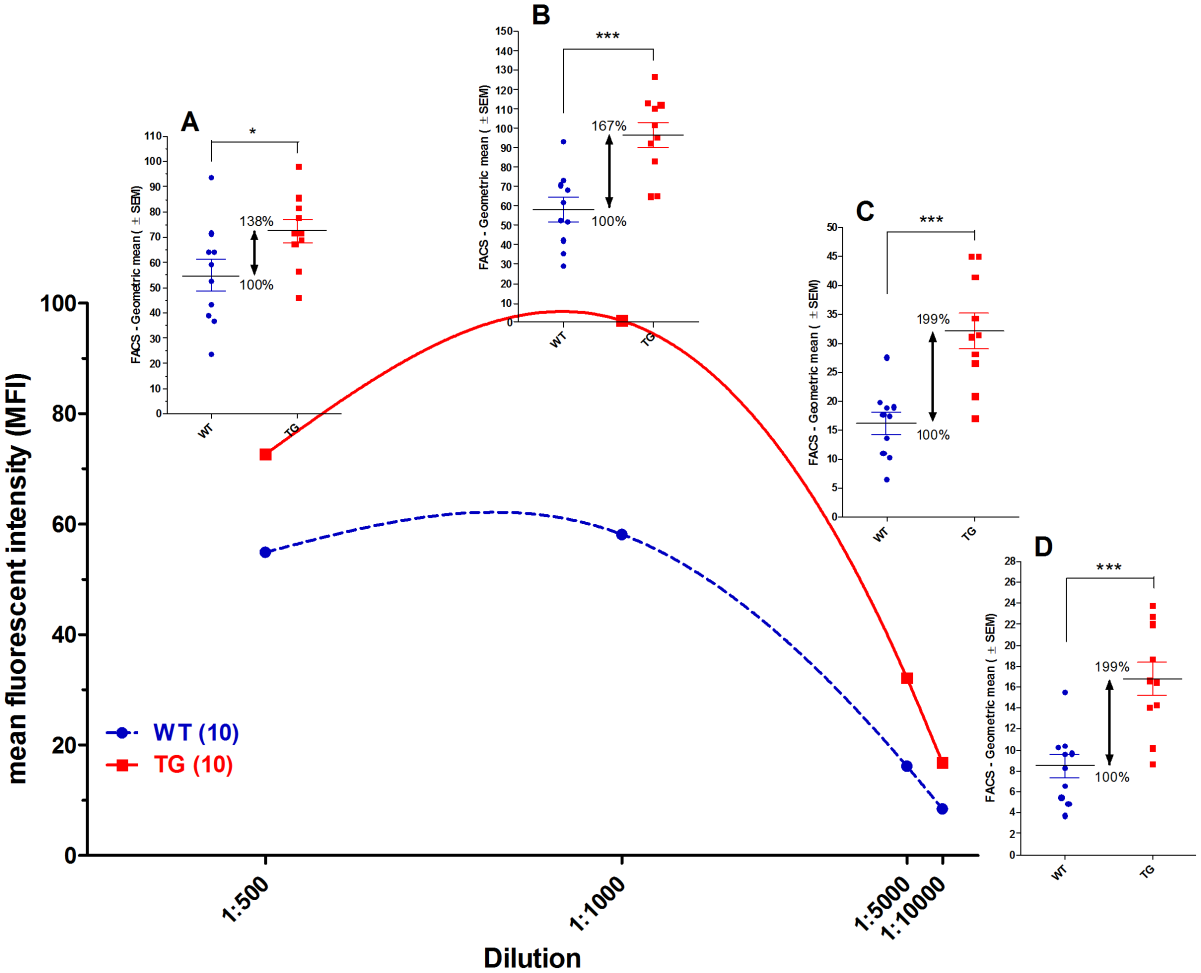
Binding of rATG to Jurkat cells from serum samples at different dilutions. Flow cytometry binding assay was performed and the geometric means of fluorescence intensities obtained are presented for different immune sera dilutions. Antigen binding of individual samples at immune serum dilutions of 1∶500 (**A**), 1∶1000 (**B**), 1∶5000 (**C**) and 1∶10000 (**D**) are shown in small inserts. Data show that Tg sera bindings were significantly higher than its wt controls in all dilutions. Each dot represents one animal as an average of three measurements (* *P*<0.05; *** *P*<0.001).

From the individual samples collected on day 21, specific antibody binding was also examined using different immune serum dilutions. Data show that the antibody binding was significantly higher in Tg rabbits at every dilution analyzed (**Figure 2**).

**Figure 3:**
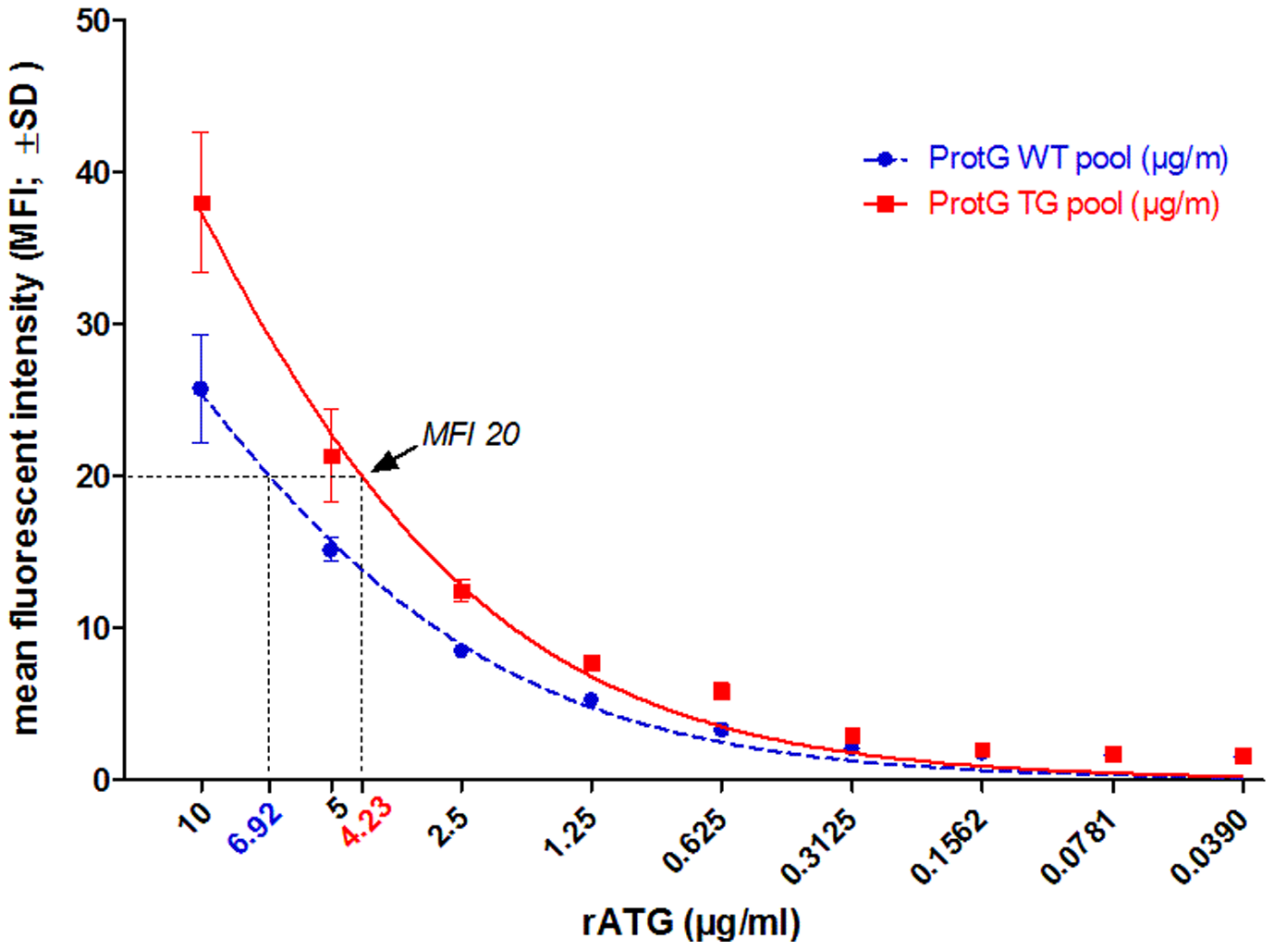
Binding of Protein-G purified, pooled Tg and wt IgG antibodies to Jurkat cells. The binding capacity of the Tg IgG analyzed by flow cytometry in a concentration range from 10 - 0.039 µg/ml was higher at all concentrations compared to their wt controls. The non-linear regression analysis indicated that binding activities of MFI 20 was achieved at concentration of 6.92 µg/ml or 4.23 µg/ml, in cases of wt or Tg preparations, respectively, indicating that Tg IgG binding was more than 60% higher than its wt control.

We also analyzed the Jurkat binding capacity of the Protein G purified total IgG preparations in the range of 10-0.039 µg/ml. At all concentrations, the binding capacity of the Tg IgG was superior compared to their wt controls. The non-linear regression analysis indicated that MFI 20 was achieved at concentrations of 6.92 µg/ml or 4.23 µg/ml, in cases of wt or Tg preparations, respectively. (We have chosen MFI 20 as this value was plotted in the linear range of the fitted curves.) Thus the IgG preparation of the Tg rabbits contains 60% more Jurkat specific IgGs compared to the IgG preparation from wt animals (**Figure 3**).

### 3. FcRn Tg rabbits produce antibody with greater complement-mediated cytotoxicity

**Figure 4:**
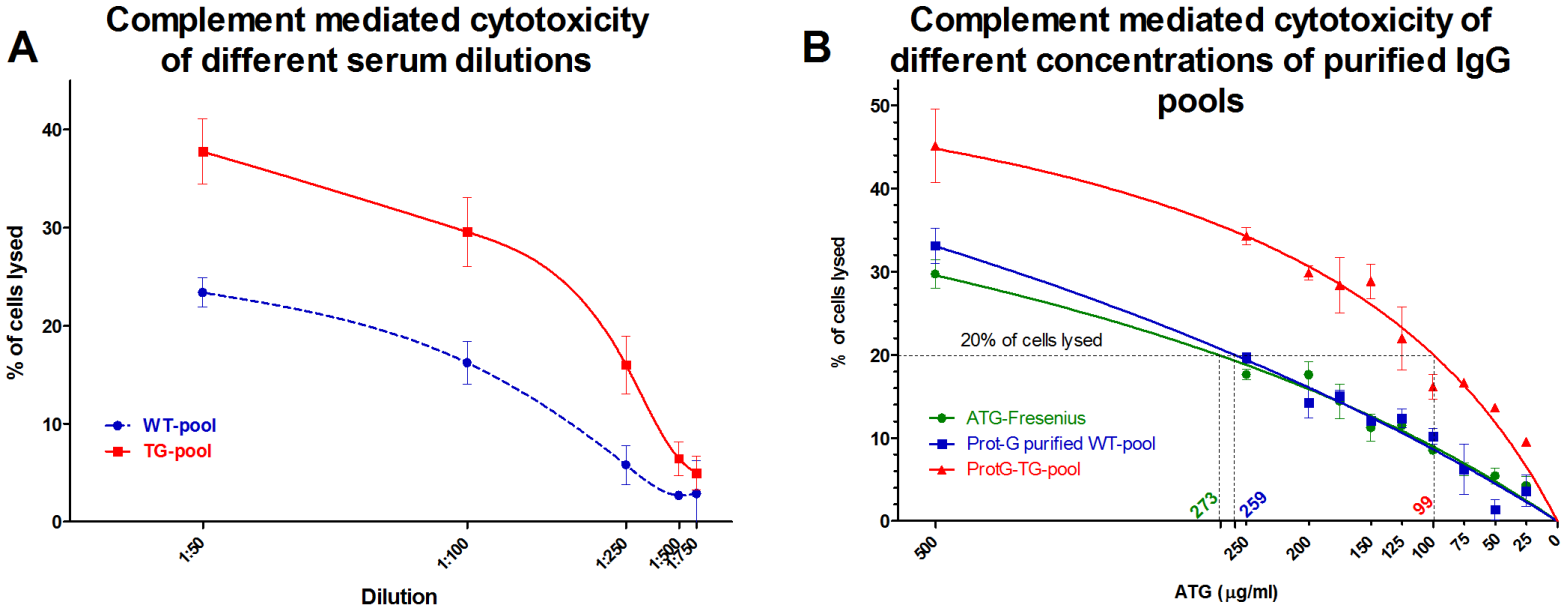
Complement activated cytotoxicity of rATG from Tg and wt rabbits. **A.** Complement activated cytotoxicity of pooled immune serum samples collected from wt and Tg rabbits on day 21 of immunization. Tg-pool of immune sera was more efficient in killing Jurkat cells at all dilutions. **B.** Concentration dependent induction of complement activated cytotoxicity by protein-G purified IgG collected on day 21 of immunization from wt and Tg rabbits (ATG-Fresenius S IgG was used as reference). We observed more efficient cytotoxicity of the Tg samples at all concentrations, and when cytotoxicity curves were analyzed with non-linear regression analysis, data showed that 20% cytotoxic activity was achieved by adding 259 µg/ml, 273 µg/ml or 99 µg/ml IgG preparations from the wt IgG, ATG-Fresenius or Tg IgG preparations, respectively, indicating that Tg IgG mediated cytotoxicity was more than 260% more efficient than its wt control. Percent of lysed cells was determined by flow cytometry analyzing propidium iodide incorporation (samples were analyzed in triplicate and error bars indicate standard deviations of these measurements).

The immunosuppressive activity of ATG has been thought to result primarily from the depletion of peripheral T lymphocytes from the circulating pool through complement-dependent lysis or activation associated apoptosis. In our experiments, we analyzed the efficiency of the pooled serum samples and purified IgG preparations by complement-mediated cytolysis. The Tg-pool of immune sera was more efficient in killing Jurkat cells at all dilutions(**Figure 4A**).

The same tendency was evident when we compared Protein G purified IgG preparations of wt and Tg rabbits and also a commercially available ATG-Fresenius preparation, as internal control. We observed more efficient complement-mediated cytotoxicity of the Tg IgG at all concentrations. 20% cytotoxic activity was achieved with 259 µg/ml, 273 µg/ml or 99 µg/ml IgG preparations from the wt rabbits, ATG-Fresenius product or FcRn Tg rabbits, respectively, based on the cytotoxicity non-linear regression analysis. ATG-Fresenius IgG showed good correlation with our wt IgG preparation, although immunization and purification protocols of ATG-Fresenius IgG differ from those we used in this study and thus direct comparative conclusion cannot be made. We concluded that the purified Tg IgG was more than twice as efficient (2.6-fold) in mediating cytotoxicity as compared to its wt control (**Figure 4B**).

### 4. FcRn Tg rabbits produce higher titers of antigen-specific IgM and IgG antibodies and many more antigen specific B cells when immunized with soluble antigens

Binding and cytotoxicity analyses from Protein G purified IgGs indicated that FcRn Tg rabbits produced more Jurkat-specific antibody as compared to their wt controls. It is well-known that FcRn prevents degradation of IgG but such protection does not distinguish between antigen-specific and antigen non-specific IgGs. As a result, the disproportionally higher amount Jurkat-specific IgG in the Tg rabbits suggests greater number of antigen(Jurkat)-specific B cell development in these animals, a feature that we observed in our FcRn Tg mice [21], [22]. To test this hypothesis, we used two different soluble proteins, ovalbumin (OVA) and human α1-anti-trypsin (hA1AT), for immunizations and analyzed the antigen-specific B-cell numbers with ELISPOT assays. (We have chosen soluble proteins to analyze the number of antigen-specific B cells with ELISPOT assay, as analyzing living Jurkat cell specific B cell number is not feasible.)

**Figure 5:**
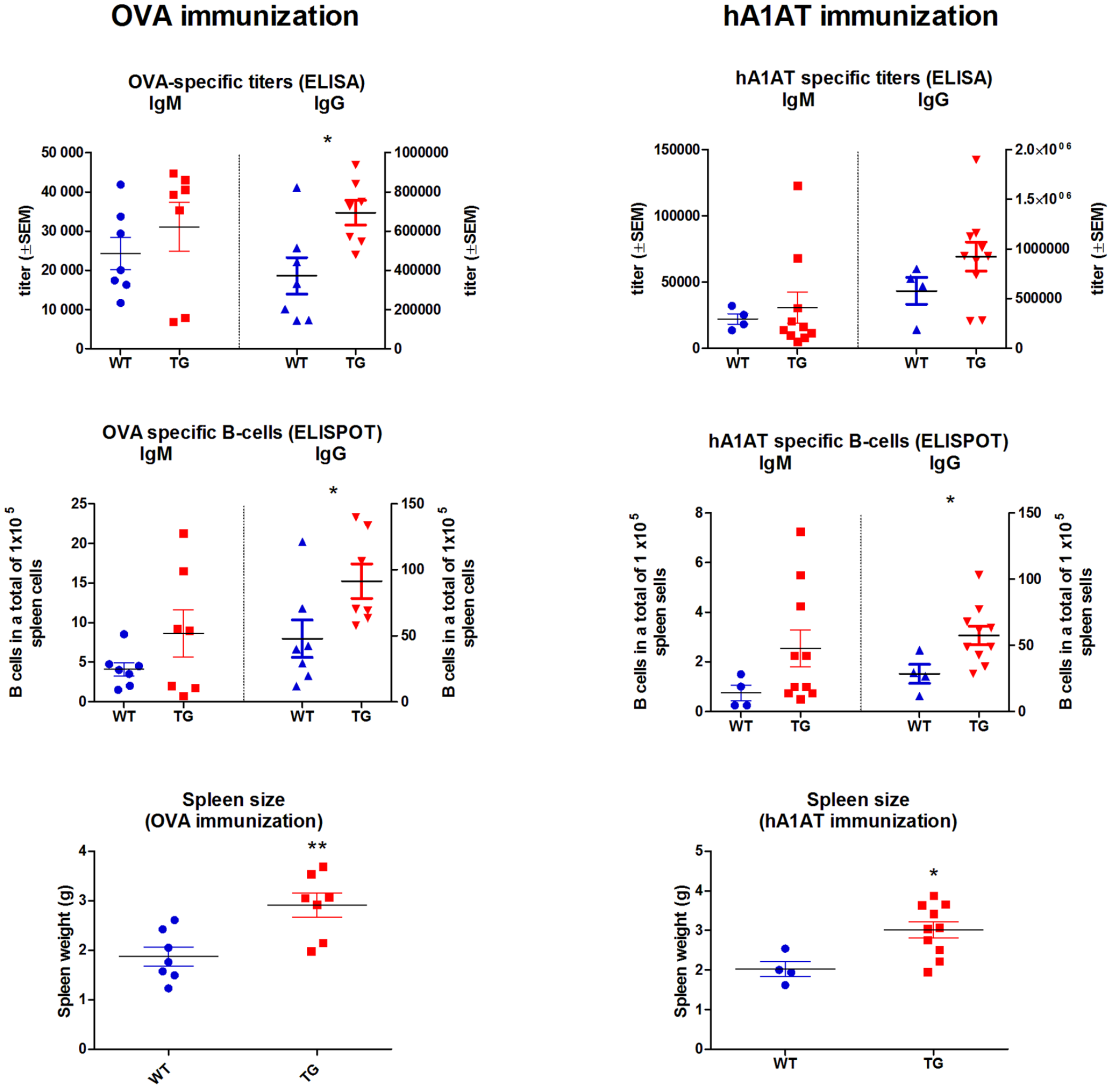
FcRn Tg rabbits have augmented antigen specific B-cell activation. Immunization with ovalbumin (OVA) and human α1-anti-trypsin (hA1AT) resulted in generally higher level IgM and IgG titers measured by ELISA, more antigen specific IgM and IgG producing B cells determined by ELISPOT, and larger spleen in Tg rabbits as compared to their wt controls (* *P*<0.05; ** P<0.01).

In both cases, FcRn Tg rabbits produced higher titers of antigen-specific IgM and IgG, showed larger spleen and produced many more antigen-specific B-cells than their wt controls (**Figure 5**). Analysis of the spleen cell composition showed a lower proportion of B and T lymphocytes in Tg animals compared to their wt controls, however calculating the total cell number, their absolute numbers were higher in Tg animals (data not shown). It has to be noticed that total spleen cells were used for ELISPOT assays, which means proportionally more antigen-specific B cells in Tg animals. These data indicate that FcRn Tg rabbits have augmented antigen specific B-cell activation as compared to their wt controls and thus the proportionally higher level of Jurkat cell specific antibodies in the purified Tg IgG preparation based on its binding capacity and complement mediated cytotoxic activity is the result of the many more activated Jurkat-specific B-cells in the Tg animals as compared to their wt controls.

## Discussion

ATG is widely used as a state of the art treatment for various clinical conditions including prevention or rescue treatment of acute rejection in organ transplantation [23], conditioning for hematopoietic stem cell transplantation, treatment of severe aplastic anemia, various autoimmune diseases, and more recently for the treatment of graft-versus-host disease [24]. ATG binds to multiple cell surface proteins expressed on T cells [25], [26]. The immunosuppressive activity of ATG has primarily been thought to result from the depletion of peripheral lymphocytes from the circulating pool through complement-dependent lysis or activation-associated apoptosis [23], [27]–[29]. Other potential mechanisms of action include modulation of surface adhesion molecules or chemokine receptor expression [30] and expansion or generation of regulatory T cells [31].

There is an increasing incidence of organ transplantations, and associated with this is a growing need for immune suppressive therapy with ATG. Hundreds of thousands of rabbits, kept in cost intensive and labor intensive specified pathogen free (SPF) conditions, are required to secure market demand. We were interested to assess if using our recently developed genetically modified rabbits that overexpress the rabbit neonatal Fc receptor (FcRn) and that generate an augmented humoral immune response [17] would favorably contribute to the production of this clinically relevant product.

FcRn is known to be involved in transporting IgGs within and across cells of diverse origin, and in doing so, they regulate concentration and transport of IgG and albumin throughout the body [32]. More recently, several publications have shown that FcRn plays major roles in antigen-IgG immune-complex phagocytosis by neutrophils [33], in antigen presentation of IgG immune complexes by professional antigen presenting cells [34]–[37] and in generating antigen specific antibodies [36]. We and others have reported previously that higher than normal expression level of FcRn reduced exogenous IgG catabolism in Tg mice, resulting in higher circulating levels of IgG [38]–[40]. Our recent studies demonstrated that FcRn overexpression in Tg mice augments antigen-specific humoral immune response with larger numbers of antigen specific B cells [21], [22], more efficient hybridoma production [22], increased diversity of induced antibodies [41] and the generation of antibodies against weakly immunogenic antigens [42].

Rabbits are important sources of polyclonal and monoclonal antibodies. We have recently reported on the development of Tg rabbits on New Zealand White genetic background that carry and express an extra copy of rabbit FcRn (when they are heterozygous) and that generate a boosted immune response when immunized with ovalbumin, TNP-KLH and KLH-conjugated peptide [17]. Our results in these Tg rabbits are consistent with the data previously generated in FcRn Tg mice that also show significantly augmented humoral immune response against various antigens, including targets with weak immunogenicity [21], [22], [42].

The presented study was undertaken to test our FcRn Tg rabbits with another type of antigen, i.e. live cells. We have chosen the human lymphoblast T-cell line (Jurkat) that is used to immunize rabbits for the generation of ATG. As mentioned before, the quantities of ATG that need to be produced at consistent qualities creates a challenge for this kind of manufacturing process. In this study, we immunized FcRn overexpressing Tg rabbits and their wild type littermates with live Jurkat cells and analyzed their immune sera with binding and complement mediated cytotoxic assays to compare their ATG production characteristics.

We could show that FcRn overexpression in Tg rabbits increases total IgG level by 60% and more significantly the antigen-specific antibody production against Jurkat cells (**Figures 1** **,** **2** **,** **3** **,** **4**). Based on our analysis, the purified Tg IgG contains 2.6 times as much rATG based on its cytotoxic activity as compared to wt IgG (**Figure 4B**). It is well-known that FcRn prevents degradation of IgG, but such protection does not distinguish between antigen-specific and antigen non-specific IgGs. As a result, the disproportionally higher amount Jurkat-specific IgG in the sera of Tg rabbits suggests greater number of antigen(Jurkat)-specific B cell development in these animals, a feature that we observed in our FcRn Tg mice [21], [22]. Since it is technically not feasible to determine the expansion of live Jurkat cell-specific B cells, we added experiments in which we immunized Tg and wt rabbits against ovalbumin and hA1AT. These experiments demonstrated a much enlarged antigen-specific B cell population in the spleen of Tg rabbits as compared to wt animals (**Figure 5**) and suggest that this mechanism contributes to the immune response in Tg rabbits against Jurkat cells, similar to the mechanisms identified in Tg mice that overexpress FcRn.

This is the first published data set that shows an increased number of antigen-specific B cell in FcRn Tg rabbits post immunization. Moreover our previous data revealed that these FcRn Tg rabbits have improved serum IgG protection [17] which further increases the amount of useful Jurkat-specific IgG.

For human therapy, especially for long-term administration, fully human form of ATG is required. Such humanized forms of ATG may be obtained from Tg animals that have disrupted endogenous immunoglobulin production and, produce human immunoglobulins. Rabbits have a short generation time, produce large numbers of offspring and can be raised under SPF condition. They have long been an important source of polyclonal and, more recently also, monoclonal antibodies [43], [44]. As a result, there is an increasing interest in raising human antibodies in these animals and efforts are ongoing to establish rabbits expressing human IgG to achieve this [45].

The responsiveness of these animals to given antigens, and subsequent production of sufficient quantities of polyclonal antibodies is a key prerequisite for the viability of these humanized systems for commercial use. Human IgG injected into the maternal circulation is transported well to the rabbit fetus [46], and its clearance is similar to rabbit IgG [47], indicating that rabbit FcRn efficiently binds human IgG. Thus, our FcRn overexpression technology could make qualitative and quantitative contributions to improvements in commercial rATG production in genetically engineered rabbits.

The results of this study show the usefulness of the technology in a medically relevant application, i.e. to overcome the limitations in production of ATG and create the opportunity for a more consistent and more potent product with a significantly improved yield per animal. Our data show that the quantity of harvestable polyclonal Jurkat-specific rATG per Tg animal is approximately 3.9 times higher (considering the 1.5 times more total IgG and the 2.6 times higher cytotoxic efficiency of the purified IgG of the Tg rabbits compared their wt controls), when using our specified methods, and thus one could estimate that the number of rabbits required to produce any given quantity of polyclonal Jurkat-specific ATG could be reduced by approximately 80%. This reduction in the size of the animal colony would enable a significant improvement in inter-animal variability and would significantly reduce complexities of manufacturing-specific QC programs.

These data provide the rationale for introducing FcRn overexpression as a major improvement into the process of animal (rabbit)-produced ATG and other polyclonal or monoclonal antibodies.
